# Significance of Misoprostol-Induced Cervical Ripening Prior to Hysteroscopy

**DOI:** 10.7759/cureus.76336

**Published:** 2024-12-24

**Authors:** Madiha Iqbal, Naheed Akhter

**Affiliations:** 1 Obstetrics and Gynaecology, Hayatabad Medical Complex Peshawar, Peshawar, PAK; 2 Obstetrics and Gynaecology, Khyber Teaching Hospital, Peshawar, PAK

**Keywords:** cervical ripening, dilation, hysteroscopy, improvement, misoprostol, procedure

## Abstract

Background

Hysteroscopy, a minimally invasive procedure for diagnosing and treating intrauterine pathologies, can be challenging due to inadequate cervical dilation, leading to procedural difficulties and patient discomfort. Misoprostol, a synthetic prostaglandin E1 analog, is increasingly used for cervical ripening to ease hysteroscopic procedures.

Objective

To evaluate the efficacy and safety of misoprostol for cervical ripening prior to hysteroscopy.

Material and methods

This prospective study included 539 women who presented to the Gynecology OPD for elective hysteroscopy. Participants were randomly assigned to either Group A (n=300), who received 400 µg misoprostol vaginally at night before hysteroscopy, or Group B (n=239), who had a placebo. The outcomes assessed were cervical dilation, procedural time, patient-reported pain during hysteroscopy, and placement of a copper IUD on post-insertion day 1 as defined by the visual analog score (VAS), incidence of cervical lacerations, or adverse side effects.

Results

Group A had significantly higher rates of achieving greater cervical dilation compared to Group B. Specifically, 50% of patients in Group A achieved a dilation of 7-8 mm, and 15% achieved dilation greater than 8 mm, whereas only 10% and 5% of patients in Group B achieved these levels of dilation, respectively. The need for additional mechanical dilation was lower in Group A (10% vs. 55%). Procedural times were shorter in Group A, with 75% of procedures taking 10 minutes or less compared to 30% in Group B. Patient-reported pain was also lower in Group A.

Conclusion

Misoprostol improves cervical ripening before hysteroscopy, reducing the need for mechanical dilation, shortening operation time, and lowering pain levels. Despite moderate side effects, it remains a key preoperative cervical preparation due to its procedural and patient comfort benefits.

## Introduction

Hysteroscopy is a surgical intervention that allows visualization of the uterine cavity and is an important tool for the diagnosis and treatment of several intrauterine conditions [1]. Although associated with advantages such as shorter recovery time and minimal scarring, hysteroscopy can be extremely painful for the patient or challenging from a procedural perspective if cervical dilation is inadequate. Proper cervical ripening is crucial for the successful and safe insertion of the hysteroscope, minimizing the risk of cervical trauma, uterine perforation, and excessive procedural pain [1,2].

Misoprostol, a synthetic prostaglandin E1 analog, is now widely used because of its efficacy in cervical ripening prior to hysteroscopy. Although approved initially for gastric ulcers, misoprostol has been used off-label in obstetrics and gynecology more than any other drug, specifically in medical abortion regimens, labor induction, and postpartum hemorrhage management [3]. Its role in cervical ripening is particularly significant due to its ability to induce softening, effacement, and dilation of the cervix, which facilitates easier and safer hysteroscopic procedures.

Many studies have reported the benefits of preoperative cervical ripening with misoprostol [4,5]. Misoprostol has been shown to increase cervical dilation and reduce the need for further mechanical dilatation with an associated decrease in the rate of cervical lacerations during hysteroscopy. Misoprostol is also associated with decreased systemic side effects compared to other ripening methods such as dinoprostone [6].

Misoprostol can be administered orally, vaginally, sublingually, or buccally. Vaginal administration is widely accepted due to its sustained release; it provides a uniform pattern of pharmacokinetics and localized targeting effect on the cervix in each route [7,8]. However, the most appropriate dose and time to use misoprostol are currently still under evaluation, as it can influence its efficiency and the occurrence of adverse effects, including cramping, bleeding, and gastrointestinal discomfort.

Beyond its practical advantages, misoprostol-induced cervical ripening is significant. Misoprostol can shorten the duration of surgery, improve patient satisfaction, and possibly even improve therapy results and diagnosis precision by enabling more seamless hysteroscopic procedures [9,10]. Its application in outpatient hysteroscopy settings further emphasizes how crucial it is to the advancement of less intrusive and more accessible gynecological treatment.

## Materials and methods

**Figure 1:**
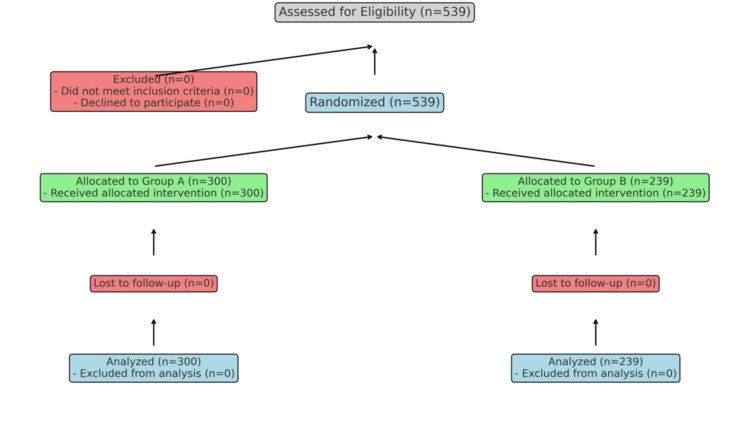
Recruitment process of participants

This prospective study was conducted in the Gynecology Department of Hayatabad Medical Complex & Khyber Teaching Hospital Peshawar from 25 August 2022 to 31 July 2024. A total of 539 women scheduled for elective hysteroscopy were enrolled (Figure 1). Eligible subjects were non-pregnant women between the ages of 18 and 45 who were confirmed to be so with a negative urine pregnancy test. Before undergoing hysteroscopy, the participants were required to have indicators for hysteroscopy (abnormal uterine bleeding/infertility as suspected pathology in utero), with no previous history of surgery or significant cervical pathologies. Exclusion criteria were: any contraindication, such as allergy to prostaglandins, active pelvic infection; severe cardiovascular, hepatic and/or renal disease, or previous uterine perforation/cervical incompetence. Approval from the Hospital Research and Ethical Committee (IREB) was obtained under reference # 860/HEC/B&PSC/2022, dated: 23-8-2022 (Appendix A). The study was also registered at "ClinicalTrials.gov (PRS "Protocol Registration & Results System") under registration number NCT06726278 on 05-12-2024 (Appendix B).

The sample size was calculated through a power analysis to detect a significant difference in cervical dilation between the Misoprostol group and the control group. Assuming a mean difference of 1 mm, a power of 80%, and a significance level of 5%, a minimum of 500 participants was required. To account for potential dropouts, 539 women were enrolled and randomly divided into two groups. Group A (n=300) received 400 µg of misoprostol vaginally 12 hours prior to the scheduled hysteroscopy while Group B (n=239) served as the control group and did not receive misoprostol. The unequal group sizes allowed for a robust evaluation of misoprostol's effects while maintaining feasibility. To ensure blinding, both misoprostol and placebo tablets were ordered separately from the hospital pharmacy and made to appear identical. Both patients and clinicians performing the hysteroscopies were blinded to group assignments.

Hysteroscopic procedure

All hysteroscopic procedures were done in the standard conditions with a 5.50 mm rigid Hysteroscope (Karl Storz, Berlin, Germany). The patients were placed in the lithotomy position, and a tenaculum was used to apply anteflexion of the cervix before insertion. The cervical dilation achieved was measured using Hegar dilators in increments of 1 mm until the hysteroscope could be inserted without resistance.

The primary outcome was the amount of cervical dilation achieved on entry to hysteroscopy. The secondary outcomes were: requirement for mechanical dilation, procedure time (from hysteroscope insertion to withdrawal), patient-reported pain score using the VAS during the procedures, an incident of cervical laceration, and adverse events, including cramping, bleeding per vagina abnormal gastrointestinal discomfort, and fever.

Data collection/analysis

Age, parity, and the reason for hysteroscopy were among the baseline demographic data gathered. Information was documented during the hysteroscopic procedure, including cervical dilatation, procedure duration, and any complications. Following the surgery, patients used the VAS to rate their level of pain. After the surgery, adverse effects were tracked and recorded for up to 24 hours. Using SPSS version 23.0 (IBM Corp., Armonk, NY, US), statistical analysis was carried out, with a significance level set at p-value ≤0.05.

## Results

In Group A, 35% of women were aged 18-30, 45% were aged 31-45, and 20% were aged 46-65. In Group B, 34% were aged 18-30, 46% were aged 31-45, and 20% were aged 46-65. In Group A, 40% were nulliparous, 45% had 1-2 previous pregnancies, and 15% had more than 2 previous pregnancies. In Group B, 41% were nulliparous, 44% had 1-2 previous pregnancies, and 15% had more than 2 previous pregnancies. In Group A, 50% of women had abnormal uterine bleeding, 32.5% had infertility, and 17.5% had suspected intrauterine pathology. In Group B, 49% had abnormal uterine bleeding, 35% had infertility, and 16% had suspected intrauterine pathology (Table 1).

**Table 1:** Baseline characteristics

Characteristic	Group A, n (%)	Group B, n (%)
18-30 years	105 (35%)	81 (34%)
31-45 years	135 (45%)	110 (46%)
46-65 years	60 (20%)	48 (20%)
Nulliparous	120 (40%)	98 (41%)
1-2	135 (45%)	105 (44%)
>2	45 (15%)	36 (15%)
Abnormal uterine bleeding	150 (50%)	117 (49%)
Infertility	98 (32.7%)	84 (35.1%)
Suspected intrauterine pathology	52 (17.3%)	38 (15.9%)

The primary outcome measured was cervical dilation at the time of hysteroscopy. Group A had significantly higher rates of achieving greater cervical dilation compared to Group B. Specifically, 50% of patients in Group A achieved a dilation of 7-8 mm, and 15% achieved dilation greater than 8 mm, whereas only 10% and 5% of patients in Group B achieved these levels of dilation, respectively (Table 2).

**Table 2:** Primary outcome Statistical test: chi-square test; Level of significance: p = ≤0.05

Outcome	Group A n (%)	Group B n (%)	p-value	χ^2^
Cervical dilatation				
< 5	30 (10%)	131 (55%)	0.002	169.03
5-6	75 (25%)	72 (30%)		
7-8	150 (50%)	24 (10%)		
> 8	45 (15%)	12 (5%)		

Secondary outcomes included the need for additional mechanical dilation, procedural time, patient-reported pain, incidence of cervical lacerations, and adverse effects. The need for additional mechanical dilation was lower in Group A (10% vs. 55%). Procedural times were shorter in Group A, with 75% of procedures taking 10 minutes or less compared to 30% in Group B. Patient-reported pain was also lower in Group A. Forty percent of patients in Group A reported pain levels of 0-3 on the VAS, compared to only 10% in Group B. Conversely, 55% of patients in Group B reported higher pain levels of 7-10 as compared to 10% in Group A (Table 3).

**Table 3:** Secondary outcomes Statistical test: chi-square test; Level of significance: p = ≤0.05

Outcome	Group A n (%)	Group B n (%)	p-value	χ^2^
Need for additional mechanical dilation				
Yes	30 (10%)	131 (55%)	0.002	118.83
No	270 (90%)	108 (45%)		
Procedural time				
≤ 10 minutes	225 (75%)	72 (30%)	0.005	47.92
> 10 minutes	75 (25%)	167 (70%)		
Patient-reported pain (VAS)				
0-3	120 (40%)	24 (10%)	0.040	74.47
4-6	150 (50%)	84 (35%)		
7-10	30 (10%)	131 (55%)		
Incidence of cervical lacerations				
Yes	8 (2.5%)	30 (12.5 %)	0.006	38.47
No	293 (97.5%)	209 (87.5%)		
Adverse effects				
Cramping	90 (30%)	48 (20%)	0.028	4.803
Bleeding	68 (22.5%)	36 (15%)	0.113	2.515
Gastrointestinal discomfort	38 (12.5%)	24 (10%)	0.451	0.567
Fever	15 (5%)	10 (4%)	0.683	0.167

The incidence of cervical lacerations was lower in Group A (2.5% vs. 12.5%). Regarding adverse effects, cramping and bleeding were more common in Group A, with cramping occurring in 30% of patients as compared to 20% in Group B. Bleeding was reported by 22.5% of patients in Group A compared to 15% in Group B (Table 3).

## Discussion

Hysteroscopy is the most common procedure in gynecology performed for diagnostic and therapeutic purposes, which includes various intrauterine pathologies such as abnormal uterine bleeding or infertility. Hysteroscopy is performed in 10-15% of all women during their lifetime [11]. However, the use of hysteroscopy may be complicated by cervical stenosis, which increases technical difficulty and procedure duration in addition to patient discomfort. The use of misoprostol, a synthetic prostaglandin E1 analog that facilitates cervical ripening, has increasingly been reported to improve the feasibility and safety of hysteroscopy [12].

In comparison to a placebo, misoprostol significantly increased cervical dilation. Our research showed that 50% of patients in Group A achieved cervical dilations of 7-8 mm, compared to only 15% of patients in Group B. Additionally, 8 mm dilation was attained by 10% of patients in Group A while only 5% of patients in Group B reached this level. This confirms the results by Wu HL et al. and Abdelhakim AM et al., who also reported that misoprostol enhanced cervical dilation in non-pregnant and premenopausal women and decreased the requirement for mechanical dilation [12,13]. Misoprostol is beneficial in encouraging cervical softening, which is important for reducing procedural complications and enhancing outcomes. This is seen by the increased cervical dilation in the misoprostol group.

Procedural time was significantly shorter in group A with 75% of procedures performed within 10 minutes as opposed to only 30% in group B, which is comparable to a study by Cheung K et al., who reported that pre-procedural misoprostol had reduced the duration of hysteroscopy [14]. Decreased time to treatment is probably a result of the enhanced cervical access and subsequently lesser need for manipulating by mechanical means with associated delays. Obvious benefits of shorter procedural times include a lower degree of patient discomfort and exposure to periprocedural complications related in part to prolonged procedures.

Significantly less patient-reported pain was a feature of the women in group A than those in group B (40% Vs 10%, p=0.041), consistent with the study by Kerr RS et al. who found that giving misoprostol prior to the procedure significantly reduces pain on hysteroscopy [15]. In contrast, it differs from the Hua Y et al. results since there were no statistically significant differences in pain scores between the misoprostol and placebo groups [16]. This may be due to variations in pain perception and anxiety level of patients or the experience of operators, which can affect pain outcomes.

Compared to group B, group A had a substantially lower incidence of cervical lacerations (2.5% versus 12.5%). Sangchai P et al., who noted that misoprostol decreased cervical damage during hysteroscopy, support this conclusion [17]. The most likely explanation is that misoprostol-induced cervical ripening lowers the risk of trauma by reducing the need for severe mechanical dilation. This decrease in cervical lacerations is a substantial benefit because these injuries can complicate rehabilitation and cause significant morbidity.

More adverse effects, such as cramping (30% vs. 20%) and bleeding (23% vs. 15%), were reported by Group A as compared to Group B. The current outcome for the use of misoprostol in cervical ripening and abortion induction was comparable to that reported by Siddique S et al. [18]. There were no differences in both groups regarding fever or gastrointestinal pain, which is consistent with the Karimzadeh A et al. study that shows these side effects are rare [19].

This study introduces a novel approach by using misoprostol for cervical ripening prior to hysteroscopy, addressing common challenges, such as cervical stenosis and procedural discomfort, which are not widely explored in previous research. The strengths of the study include a well-structured methodology with rigorous inclusion and exclusion criteria, ensuring the relevance and reliability of the findings. A relatively large sample size and comprehensive outcome measures, including cervical dilation, procedural time, and patient-reported pain, add robustness to the study's conclusions.

However, limitations include the lack of randomization and blinding, which may introduce bias. The short follow-up period limits the assessment of long-term outcomes, and variability in clinicians’ techniques and subjective pain reporting may affect consistency. A larger, multicenter trial could further validate these findings and increase their applicability.

## Conclusions

Misoprostol significantly improves cervical ripening before hysteroscopy, which reduces the need for additional mechanical dilatation, shortens operation time, and lowers patient-reported pain levels. Despite having a higher risk of moderate side effects, including cramping and bleeding, misoprostol is routinely used in clinical practice for cervical preparation prior to hysteroscopy because of its overall benefits in enhancing procedural outcomes and patient comfort.

## Figures and Tables

**Figure 2:**
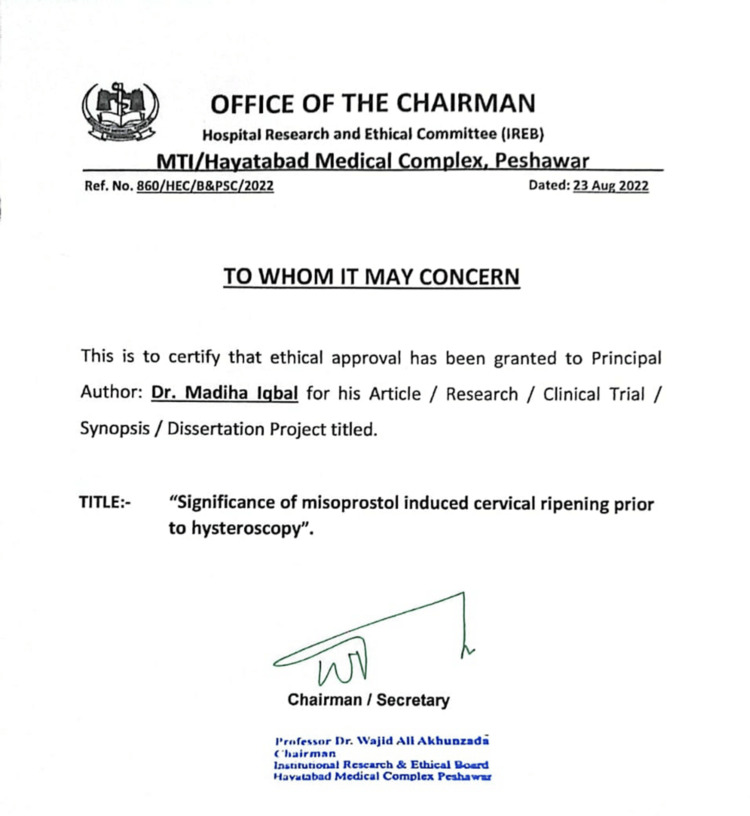
Ethical approval certificate

**Figure 3:**
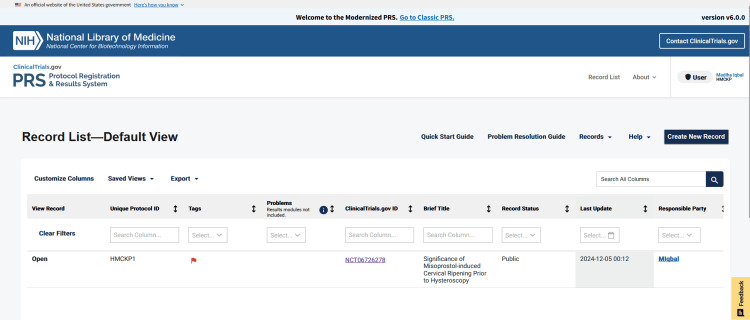
Clinical trial registration

## Appendices

Appendix A 

Appendix B 
